# Combining DL-Methionine and *Bacillus* *thuringiensis* Subspecies *israelensis*: Prospects for a Mosquito Larvicide

**DOI:** 10.3390/insects11120880

**Published:** 2020-12-11

**Authors:** Elise A. Richardson, Nicole O. Abruzzo, Caitlin E. Taylor, Bruce R. Stevens, James P. Cuda, Emma N. I. Weeks

**Affiliations:** 1 UF/IFAS Entomology and Nematology Department, University of Florida, Gainesville, FL 32611, USA; ear6296@ufl.edu (E.A.R.); nabruzzo@ufl.edu (N.O.A.); llamataylor@ufl.edu (C.E.T.); jcuda@ufl.edu (J.P.C.); 2 Department of Physiology and Functional Genomics, University of Florida, Gainesville, FL 32603, USA; stevensb@ufl.edu

**Keywords:** *Anopheles quadrimaculatus*, *Aedes aegypti*, larvae, pesticide, antagonism, synergism

## Abstract

**Simple Summary:**

With the increasing threat that mosquito borne diseases pose to public health, the demand for environmentally sustainable pesticides has been increasing in recent years. Pesticides that target the larval stage (i.e., larvicides) are particularly useful for controlling mosquito populations as they strike at the source. Currently, *Bacillus thuringiensis* subspecies *israelensis* (BTI) is a commonly used mosquito larvicide but some studies show signs of resistance development. DL-methionine is an essential amino acid that has mosquito larvicidal capabilities, while also having minimal negative effects on non-target organisms in laboratory experiments. In this study, our objective was to evaluate the effect of these two larvicides individually and together at reducing mosquito survival. We found that while DL-methionine was more toxic to *Anopheles quadrimaculatus* than *Aedes aegypti*, the opposite was true for BTI. Additionally, when the combination was tested against *An. quadrimaculatus* larvae at higher concentrations the active ingredients were complementary and the effect was equal to both ingredients alone. However, the active ingredients were antagonistic when tested against *Ae. aegypti* larvae. These findings are important as they show the potential for DL-methionine and the combination of DL-methionine with BTI to be used as a larvicide against *Anopheles* mosquitoes, which are responsible for transmitting malaria.

**Abstract:**

Mosquito larvicides can reduce mosquito populations at the source, potentially decreasing biting rates and pathogen transmission. However, there is a growing need for mosquito larvicides that are environmentally sustainable. *Bacillus thuringiensis* subspecies *israelensis* (BTI) is a naturally occurring bacterium commonly used as a larvicide to manage mosquito populations. Methionine is an essential amino acid that has demonstrated toxic properties against larval mosquitoes in laboratory experiments, while having minimal effects on non-target organisms. The goal of this study was to evaluate the potential for a novel combination larvicide by testing for compatibility between these two active ingredients. We began by determining the lethal concentration values (LCs) of BTI and DL-methionine against *Anopheles quadrimaculatus* Say and *Aedes aegypti* Linnaeus (Diptera: Culicidae) larvae. These bioassays were conducted in glass jars and mortality was observed 48 h post-treatment. We found that while DL-methionine was more toxic to *An. quadrimaculatus* than *Ae. aegypti*, the opposite was true for BTI. Then, we used these LCs to conduct bioassays with a combination of BTI and DL-methionine to determine the relationship between the two active ingredients when used against *An. quadrimaculatus* and *Ae. aegypti* larvae. The findings of this study demonstrate that BTI and DL-methionine have the potential to be complementary due to their additive properties at higher concentrations and effect levels when tested against *An. quadrimaculatus*. However, an antagonistic relationship was detected at the concentrations tested with *Ae. aegypti.* These results are encouraging and imply that a DL-methionine or BTI/DL-methionine combination larvicide could be used in management of *Anopheles* species.

## 1. Introduction

Mosquitoes (Diptera: Culicidae) are responsible for the transmission of pathogens that cause life-threatening diseases in humans, livestock, pets, and wildlife [1]. For example, *Anopheles* spp. are the vectors of *Plasmodium* spp., the protozoans that cause malaria. In 2016, malaria was responsible for about 445,000 human deaths out of 216 million cases [2]. In addition, *Aedes* spp. transmit multiple arboviruses of importance throughout the world including Zika, yellow fever, dengue, and chikungunya viruses [3]. 

Mosquito larvicide applications in potential development sites can be a highly effective method for mosquito population control as they kill the larvae before they are able to complete their development and contribute to pathogen transmission. Currently, mosquito larval populations are controlled using organophosphates, insect growth regulators (IGRs), and microbial control agents [4], but all have disadvantages. For example, the organophosphate temephos, which was registered for mosquito control in the USA, is currently undergoing cancellation. In 2017, the U.S. Environmental Protection Agency (EPA) ruled that public/private mosquito control agencies that had previously obtained temephos stocks could only continue using them for mosquito control until the supply is exhausted [5]. IGRs are effective, but their toxicity is not limited to mosquitoes; non-target effects on other Diptera including chironomids have been reported [6]. However, the effects of the commonly used mosquito control active ingredient S-methoprene on mammals, fish, birds, and bees are minimal especially at the concentrations necessary for mosquito control [6]. Unfortunately, following treatment failures, resistance in field populations to S-methoprene has been reported [7]. Microbial agents currently used for controlling larval mosquitoes are *Bacillus thuringiensis* subspecies *israelensis* (BTI) and *Lysinibacillus sphaericus* (previously *Bacillus sphaericus*) [8]. *Bacillus thuringiensis* is a naturally occurring bacterium that can be found in soils [8], the various subspecies are specific to different insect groups. These bacteria express Cry and Cyt proteins that target the aminopeptides residing in epithelial apical membranes of insects with highly alkaline pH midgut physiology and are insecticidal by imparting leakiness to the epithelium [8]. Both species are more specific than IGRs, however, BTI has direct effects on other nematoceran Diptera, including black flies and midges (biting and non-biting). While direct application of BTI has been shown to be safe for non-target organisms including vertebrates and most invertebrates [9,10,11], direct effects on nematoceran Diptera can lead to indirect effects on the food web due to the role of these insects in the diet of many insectivorous vertebrates [12]. Furthermore, there have been multiple publications providing evidence of resistance or the potential for development of resistance to BTI and *Lysinibacillus sphaericus* in mosquitoes [13,14,15]. This information illustrates the importance and urgency for the development of novel pesticides to selectively control mosquito populations without impacting the environment.

Methionine is a naturally occurring nutrient—indeed it is an essential amino acid for humans and is used worldwide as a feed supplement nutrient for aquaculture and livestock. Members of our group made the serendipitous initial discovery and characterization of methionine as an alkaline midgut pesticide [16,17], followed by our quantifying its toxic properties against larval mosquitoes in laboratory-setting experiments [18,19,20], with the DL-methionine enantiomer, D-, and L-stereoisomers nearly equal in efficacy. We employed membrane biophysics coupled with site-directed mutagenesis molecular genetics experiments to demonstrate that mechanistically methionine disrupts OH^−^ and K^+^ homeostasis in the highly alkaline environment of the midgut [16,17,21,22]. These studies resulted in our cloning of the CAATCH1 gene, which is the methionine-blocked ion-coupled amino acid transporter expressed in midgut epithelium apical membranes, and is responsible for the empirically observed insecticidal activity of methionine [21,22]. BTI Cry and Cyt crystals target the same alkaline midgut epithelial membranes, but their mechanism of imparting leakiness through holes is entirely different from methionine disrupting electrolyte physiology of this midgut region. Methionine offers many desirable qualities for an effective, biorational pesticide, including its minimal effect on non-target species [18,23]. Furthermore, due to its status as an essential amino acid that must be consumed in the diet of many organisms, including mosquitoes, it is less likely that resistance will develop in the future. Previous studies have shown both BTI and methionine are independently effective mosquito larvicides [18,19,20,24]. Thus, their different mechanistic modes of action provide an ideal strategic rationale for dual application, because (1) combined at low concentrations they could enhance the disruption of midgut function, and (2) their independent mechanisms greatly reduce the probability of insect resistance from simultaneous adaptive mutations. When two active ingredients (AIs) are combined there are three potential outcomes: a synergistic, additive, or antagonistic effect. When the effect is synergistic the AIs work together to produce an enhanced effect greater than the sum of their individual actions [25]. When the effect is additive it is the sum of the AIs individual actions and when it is antagonistic it is less than the sum of the AIs individual actions. Therefore, combining AIs with an additive or a synergistic relationship can result in a complementary effect. Such a combination offers greater levels of pest control at lower concentrations. Indeed, this is a major objective of pest and resistance management.

We hypothesized that the combination of BTI with methionine would enhance mosquito larvicidal activity greater than either agent alone (with BTI alone acting as a positive control), and greater than the negative control (water), with an eye towards improving mosquitocidal efficacy and sustainability. To this end, we designed experiments to determine the relationship of DL-methionine applied with BTI in two mosquito species, *Anopheles quadrimaculatus* Say and *Aedes aegypti* L. The results demonstrated that while DL-methionine was more toxic to *An. quadrimaculatus* than *Ae. aegypti*, the opposite was true for BTI. Furthermore, although antagonistic at lower concentrations, BTI and DL-methionine were additive when tested with *Anopheles* at higher concentrations. In contrast, antagonism between the AIs was observed at the concentrations tested with *Aedes*.

## 2. Materials and Methods

### 2.1. Mosquitoes

The mosquitoes used in the study were laboratory reared individuals from strains that have been in colony for more than 20 years without any pesticide exposure. *Anopheles quadrimaculatus* were acquired as recently harvested and dried eggs in a ≃3.7 mL vial (1 dram) from the Center for Medical, Agricultural, and Veterinary Entomology (CMAVE) at the United States Department of Agriculture, Agricultural Research Service (USDA-ARS), Gainesville, Florida. The eggs were placed in 470 mL (16 oz.) plastic cups containing well water following USDA-ARS protocols [26]. The cups were placed inside an incubator held at 30 °C, 80% relative humidity (RH) and 12:12 (L:D). Once the larvae hatched, they were transferred into trays containing well water and placed inside the incubator until the larvae were 3rd instars. 

*Aedes aegypti* were obtained as dried dormant eggs on oviposition paper from the Veterinary Entomology Laboratory at the University of Florida (UF) Entomology and Nematology Department, Gainesville, Florida. Larvae were hatched in a 470 mL (16 oz.) plastic cup of deionized water for approximately one hour and then were moved to a tray of deionized water that was placed inside the incubator at the conditions described above. The larvae were kept in the tray for approximately two days, until they progressed to 2nd instars.

Larvae of both species were fed one scoop of ground tropical fish food (0.3 g, TetraMin^®^ Tropical Fish Flakes, Tetra, Blacksburg, VA, USA) the day after they hatched and were not fed again until they were placed in the jars. 

### 2.2. Bioassay

Bioassays were conducted in 946 mL (one quart) glass jars containing 500 mL of test solution. Ten to 15 *An. quadrimaculatus* or *Ae. aegypti* larvae were placed in each jar. To avoid handling-induced mortality the numbers were permitted to vary but the total count per jar was recorded once all the larvae were in the jars. Experiments conducted with *Anopheles* and *Aedes* were completed in well water and deionized water, respectively, following species-specific rearing procedures. While in the jars, the larvae were fed one pinch (≃0.05 g) of fish food (TetraMin^®^ Tropical Fish Flakes, Tetra, Blacksburg, VA, USA) daily. Assays were completed in temperature controlled environmental cabinets that were held at 30 °C, 85% RH, and 12:12 (L:D) at the UF Entomology and Nematology Department. Mortality counts were conducted after 48-h exposure. Larvae were considered dead if no movement was detected after the jars were swirled in a circular motion and if the larvae became darker in color. For the concentration response experiments, there were four jars for each treatment in each replicate. For the combination experiment, there were three (*An. quadrimaculatus*) or two (*Ae. aegypti*) jars for each treatment in each replicate due to the high number of treatments and limited space in the environmental cabinet.

### 2.3. BTI Concentration Response

BTI (*Bacillus thuringiensis* subspecies *israelensis*) was obtained as a sample of Aquabac^®^ (Aquabac^®^ XT; Becker Microbial Products Inc., Parkland, FL, USA). It had a label rate of 0.25–2.00 pts/acre and consisted of 8.0% BTI solids, spores, and insecticidal toxin particles (equivalent to 1.6 × 10^12^ International Toxin Units/L (ITU/L)). The BTI sample was used to create a stock solution concentration defined as 1×, which was equal to 0.23 mL/m^2^ or 2 pts/acre. The stock solution was prepared fresh for each replicate. Each jar containing 500 mL of solution had a diameter at the water level of 9 cm. Therefore, the water surface area was 0.0064 m^2^. To obtain the 1× label rate of 0.23 mL/m^2^, 1.5 µL of Aquabac^®^ XT was added to each jar. The resulting 500 mL of 240 µg/L Aquabac^®^ XT test solution was comprised of 2.4 × 10^5^% BTI particle solids (4.8 × 10^7^ ITU). Based on preliminary assays, concentrations were chosen to ensure mortality between 25% and 100% to calculate the concentration response curves. *Anopheles quadrimaculatus* were tested at 2.4, 4.8, 7.2. 9.6, 12.0, 24.0, 120.0, 240.0, and 1200.0 µg/L (1–5 replicates, 1300 mosquitoes). *Aedes aegypti* were tested at 2.4, 4.8, 6.0, 7.2, 9.6, 12.0, and 24.0 µg/L (1–7 replicates, 1579 mosquitoes). The water diluent (deionized or well water) depended upon the species being tested. The concentrations were made in 2 L solutions, which were divided evenly between four treatment jars (500 mL per jar). During the process of dividing up the solution amongst four jars, it was stirred constantly to ensure an even distribution of BTI to water. A negative control consisting of four jars of 500 mL of the corresponding water type was completed for each species during each replicate.

### 2.4. Methionine Concentration Response

The highest concentration to be tested, 1.00%, of DL-methionine (≥99.0%, CAS Reg. No.59-51-8; Sigma Aldrich, St. Louis, MO, USA) was prepared and stirred using a magnetic stirrer on a hot plate with the heat function turned off. This solution was prepared fresh for each replicate. Based on preliminary assays, the 1% solution was diluted with deionized water to ensure mortality between 25% and 100% to calculate the concentration response curves. *Aedes aegypti* were tested at 0.0, 1.0, 2.5, 5.0, 7.5, and 10.0 g/L (4 replicates, 1198 mosquitoes). While dividing up the solution amongst four jars, the solution was stirred constantly to ensure an even distribution of DL-methionine to water. A negative control consisting of four jars of 500 mL of deionized water was completed during each replicate.

### 2.5. Combination Experiment

To determine if the two AIs were synergistic, additive, or antagonistic, we completed a constant-ratio diagonal design Latin square for two reagent combinations [27,28]. The LC_50_ values calculated in the earlier concentration responses and from previous research for *An. quadrimaculatus* and DL-methionine [19] were used to define the concentrations for this experiment. All solutions were prepared as described in Section 2.3 and Section 2.4 with fresh stock solutions and dilutions of both AI for each replicate. For the two AIs we tested 0.25X, 0.50X, 1.00X, 2.00X, and 4.00X the LC_50_. For DL-methionine with *An. quadrimaculatus* these concentrations were: 0.2025, 0.4050, 0.8100, 1.6200, and 3.2400 g/L. For BTI with *An. quadrimaculatus* these concentrations were 21.875, 43.750, 87.500, 175.000, and 350.000 µg/L. For DL-methionine with *Ae. aegypti* these concentrations were: 0.835, 1.670, 3.340, 6.680, and 13.360 g/L. For BTI with *Ae aegypti* these concentrations were: 1.2, 2.4, 4.8, 9.6, and 19.2 µg/L. For the combination, the two AIs were mixed at a constant ratio resulting in solutions with concentrations of: 0.25×, 0.50×, 1.00×, 2.00×, and 4.00× the LC_50_ of each AI. *Anopheles quadrimaculatus* (4 replicates, 2529 mosquitoes) and *Ae. aegypti* (6 replicates, 2370 mosquitoes) were tested in well and deionized water, respectively. A negative control consisting of the same number of jars of 500 mL of the corresponding water type was completed for each species during each replicate.

### 2.6. Statistical Analysis

Mortality data (48 h exposure) from the concentration response experiments for each jar were combined by replicate and analyzed using probit as a binary response with one explanatory variable in PoloPlus (PoloPlus 1.0; LeOra Software, El Cerrito, CA, USA) [29]. Control mortality was not corrected and was used to calculate the natural response slope and standard error (Table 1). Concentration response curves were plotted and the lethal concentration to 50%, 90%, and 99% mortality (LC_50_, LC_90_, and LC_99_) was calculated. Values were only considered to be valid if the t-ratio indicated that the regression was significant (>1.96), and the heterogeneity factor indicated a good fit to the model. Residual plots were examined for outliers.

The 48-h LC_50_ values calculated above for *An. quadrimaculatus* with BTI and *Ae. aegypti* with DL-methionine and BTI were used to calculate the concentrations to be tested in the combination experiment. Additionally, the LC_50_ (0.081%) for 3rd instar *An. quadrimaculatus* with DL-methionine was extracted from Weeks et al. [19], converted to the appropriate units (0.81 g/L), and incorporated. The data in Weeks et al. [19] were collected using the same mosquito colony and following the same assay protocol.

Mortality data (48 h exposure) from the synergist experiments for each jar were combined and then averaged across replicates. Average mortality data of the AIs alone and in combination for each species were analyzed in Compusyn (ComboSyn Inc., New York, NY, USA) to determine the relationship between DL-methionine and BTI. The software takes proportion mortality data at each concentration, or the lethality fraction affected by the concentration (*f_a_*), and computes the combination indices (*CI*) using the Chou–Talalay equation and concentration-effect curve parameters [27,28]. Data were first fitted for each individual AI, BTI or DL-methionine, acting alone using Equation (1). Then, *C_m_* and *m* were determined from median-effects plots using Equation (2).

(1) $$C_{x}=C_{m}\cdot {\left[\frac{f_{a}}{1-f_{a}}\right]}^{1/m}$$

(2) $$x=\log\left(C\right)vs.\;y=\log\left(\frac{f_{a}}{f_{u}}\right)$$

where

$C_{x}$ is the concentration of each individual AI (BTI or DL-methionine);
$C_{m}$ is the median-effect concentration (i.e., LC_50_ giving 50% larval lethality);
*m* is the slope (coefficient signifying the relative sigmoidicity shape of the concentration-effect, whereby *m* = 1, >1, and <1 indicate hyperbolic, sigmoidal, or flat curves, respectively);
$f_{a}$ is the lethality fraction affected by concentration *C* (i.e., percent inhibition/100);
$f_{u}$ is the lethality fraction (i.e., $f_{a}=1-f_{u}$ ) unaffected by the concentration *C*.

Conformity to a linear relationship was assessed by correlation coefficient. Next, the Combination Index (*CI*) was determined from the Chou–Talalay equation [28], which assesses the synergism or antagonism for combinations of BTI plus DL-methionine giving mosquito larval proportion mortality. For our study involving two AIs (BTI and DL-methionine) the Chou–Talalay equation was reduced to Equation (3).

(3) $$\begin{array}{ll} CI & =\left[\frac{{\left(C\right)}_{BTI}}{{\left(C_{x}\right)}_{BTI}}+\frac{{\left(C\right)}_{Met}}{{\left(C_{x}\right)}_{Met}}\right]\\ & =\frac{{\left(C_{X}\right)}_{BTI,Met}\cdot P/\left(P+Q\right)}{{\left(C_{m}\right)}_{BTI}\cdot {\left[\frac{f_{a}}{1-f_{a}}\right]}^{1/m_{BTI}}}+\frac{{\left(C_{X}\right)}_{BTI,Met}\cdot Q/\left(P+Q\right)}{{\left(C_{m}\right)}_{Met}\cdot {\left[\frac{f_{a}}{1-f_{a}}\right]}^{1/m_{Met}}} \end{array}$$

where

$\left(C_{BTI}\right)={\left(C_{X}\right)}_{BTI,Met}\cdot P/\left(P+Q\right)$;
$\left(C_{Met}\right)={\left(C_{X}\right)}_{BTI,Met}\cdot Q/\left(P+Q\right)$;
${\left(C_{x}\right)}_{BTI,Met}={\left(C\right)}_{BTI}+{\left(C\right)}_{Met}$;
${\left(C\right)}_{BTI}/{\left(C\right)}_{Met}=P/Q$.

Relative degree of synergism is reflected in *CI*, whereby *CI* < 0.1 is very strong synergism, 0.1–0.3 is strong synergism, 0.3–0.7 is synergism, 0.70–0.85 is moderate synergism, 0.85–0.90 is slight synergism, 0.90–1.10 is nearly additive, 1.10–120 is slight antagonism, 1.20–1.45 is moderate antagonism, 1.45–3.30 is antagonism, 3.30–10.00 is strong antagonism, and >10 is very strong antagonism [30]. For each mosquito species, sequential deletion analysis (S.D.A.) was completed by iterative sequential deletion of one concentration at a time followed by calculation of the *CI* [30]. The mean and 95% confidence intervals can then be calculated at each lethality fraction. Isobolograms were prepared for each mosquito species by plotting the concentration of DL-methionine on the y-axis and BTI on the axis. When a line is drawn between the LC_50_ of each AI on its respective axis, the position of the data point for the combined treatment indicates synergistic (below the line), additive (on the line), or antagonistic (above the line) effects. 

Concentration reduction indices (*CRI*) were calculated from the reciprocal of each term in Equation (3) at the lethality fractions achieved by the combined AI mixture (Equations (4) to (6)).

(4) $${\left(CRI\right)}_{BTI}=\frac{{\left(C_{X}\right)}_{BTI}}{{\left(C\right)}_{BTI}}$$

(5) $${\left(CRI\right)}_{Met}=\frac{{\left(C_{X}\right)}_{Met}}{{\left(C\right)}_{Met}}$$

(6) $${\left(CRI\right)}_{BTI,\;Met}=\left[\frac{{\left(C_{x}\right)}_{BTI}}{{\left(C\right)}_{BTI}}+\frac{{\left(C_{x}\right)}_{Met}}{{\left(C\right)}_{Met}}\right]$$

where ${\left(C_{x}\right)}_{BTI}$ and ${\left(C_{x}\right)}_{Met}$ alone each inhibit x% and where the combination $C_{BTI,Met}=\left[C_{BTI}+C_{Met}\right]$ inhibits x%. *CRI* < 1 is unfavorable concentration reduction, *CRI* > 1 is favorable concentration reduction, *CRI* = 1 no concentration reduction.

## 3. Results

### 3.1. Individual Concentration Responses

**Figure 1:**
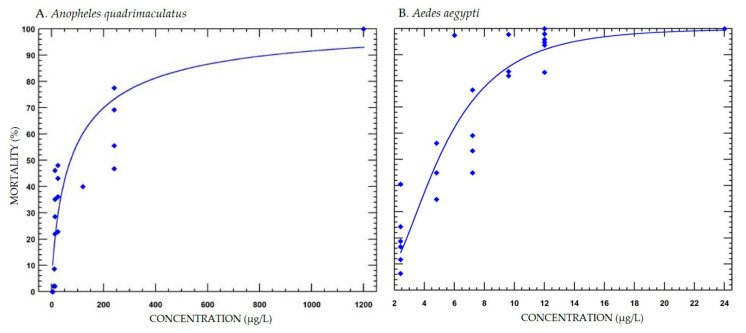
Fitted linear concentration response curves of mortality percentages in *Anopheles quadrimaculatus* 3rd instar larvae ((**A**), n = 1–5) *and Aedes aegypti* 2nd instar larvae ((**B**), n = 1–7) exposed to *Bacillus thuringiensis* subspecies *israelensis* for 48 h in glass jars. Each data point represents a replicate with the four jars combined. Curves plotted with PoloPlus (PoloPlus 1.0; LeOra Software, El Cerrito, CA, USA).

Mortality in *Ae. aegypti* and *An. quadrimaculatus* due to BTI followed a concentration response, with increasing mortality at increasing concentrations (Figure 1, Tables S1 and S2). *Aedes aegypti* larval sensitivity (i.e., concentration-response slopes) to BTI was greater as indicated by a steeper slope compared with *An. quadrimaculatus* (Figure 1, Table 1). The lethal concentration to 50% mortality for *An. quadrimaculatus* was 17.5-fold higher at 87.63 µg/L (45.93–167.70 µg/L) than that for *Ae. aegypti* at 5.02 µg/L (4.23–5.74 µg/L) (Table 1). The control mortality in these experiments were 7.99 ± 1.49% for *An. quadrimaculatus* and 2.74 ± 1.37% for *Ae. aegypti*. 

**Figure 2:**
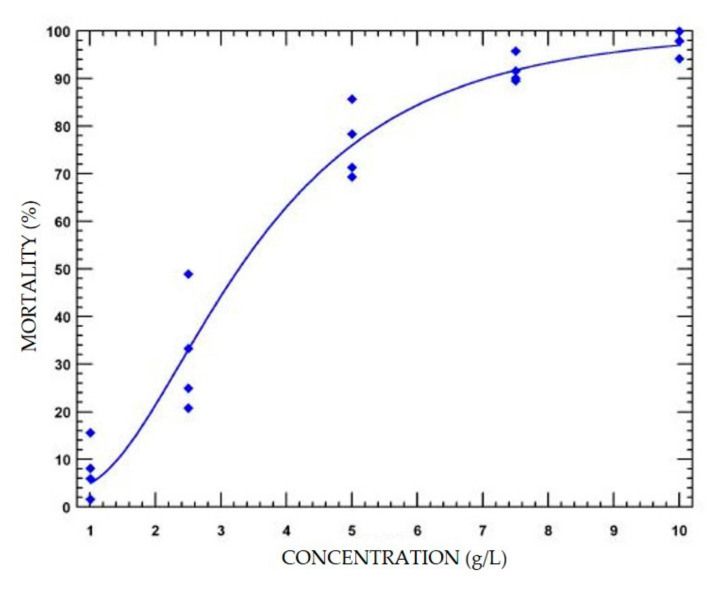
Fitted linear concentration response curve of mortality percentages in *Aedes aegypti* 2nd instar larvae (n = 4) exposed to DL-methionine for 48-h in glass jars. Each data point represents a replicate with the four jars combined. Curve plotted with PoloPlus (PoloPlus 1.0; LeOra Software, El Cerrito, CA, USA).

Methionine induced mortality in *Ae. aegypti* also increased with increasing concentration (Figure 2, Table S3). The LC_50_ of DL-methionine with *Ae. aegypti* was 3.338 g/L (2.869–3.748 g/L), which is 4-fold higher than that reported by Weeks et al. [19] and utilized in this study for *An. quadrimaculatus* (0.81 g/L, 0.77–0.85 g/L) (Table 1). The control mortality for *Ae. aegypti* in this experiment was 3.04 ± 1.27%.

### 3.2. Combination Experiment

**Figure 3:**
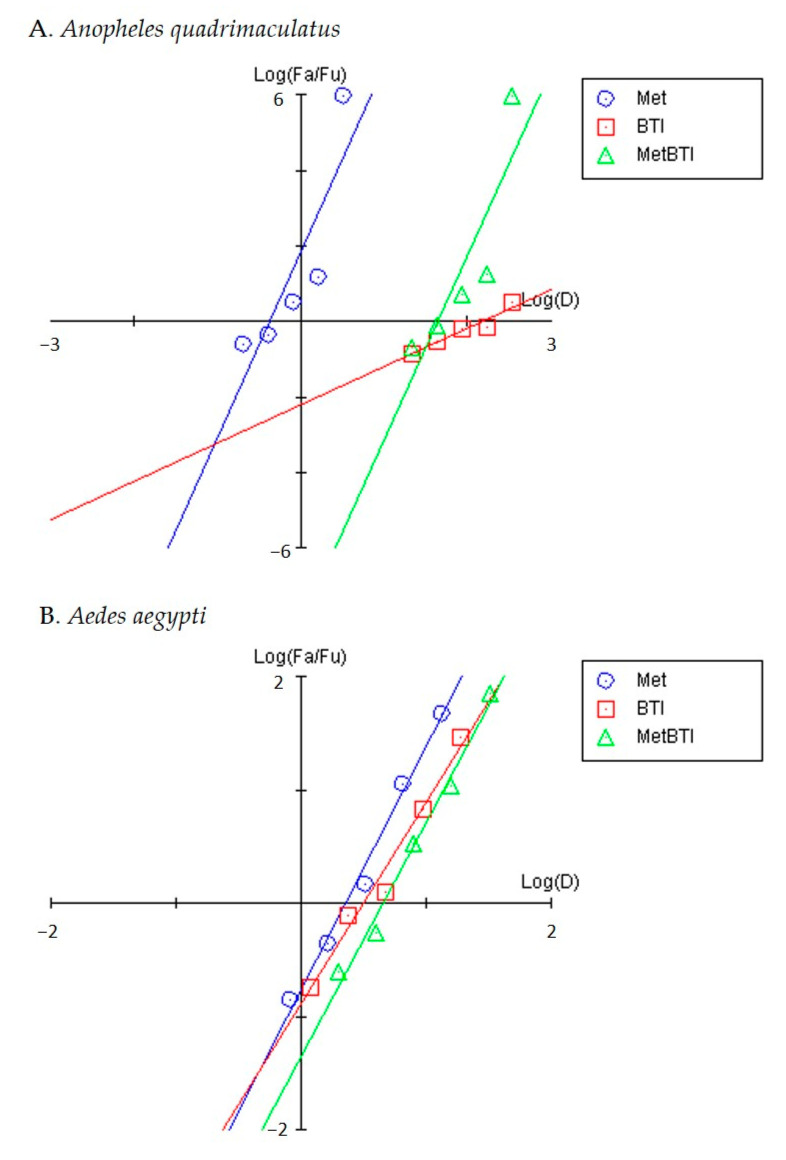
Median effect plots for *Anopheles quadrimaculatus* ((**A**), n = 4) and *Aedes aegypti* ((**B**), n = 6) for DL-methionine (Met; g/L) and *Bacillus thuringiensis* subspecies *israelensis* (BTI; µg/L) alone and in combination (MetBTI; no units). Exposures occurred over 48 h in glass jars. Ordinate values represent log of the lethality fraction (log(*f*_a_*/f*_u_)) affected by the concentration at various abscissa log concentrations (log(D)). Each data point represents the average mortality in all replicates. Plotted using the software Compusyn [30].

For BTI, DL-methionine, and the combination, *R*-values were >0.86 and >0.98 for *An. quadrimaculatus* and *Ae. aegypti* indicating a relatively good and excellent fit to the model, respectively (Table 2). For each AI alone and in combination there was increasing mortality with increasing concentration for both species (Figure 3, Tables S4 and S5). The slopes of the median effect plots were >1 indicating sigmoidal concentration response curves for all treatments with both species (Table 2). Comparison of the slopes to evaluate larval sensitivity in *Ae. aegypti* revealed similar sensitivity to each AI and the combination (Table 2, Figure 3). However, *An. quadrimaculatus* larval sensitivity to DL-methionine and the combination was greater than to BTI as indicated by a 4-fold steeper slope (Table 2).

**Figure 4:**
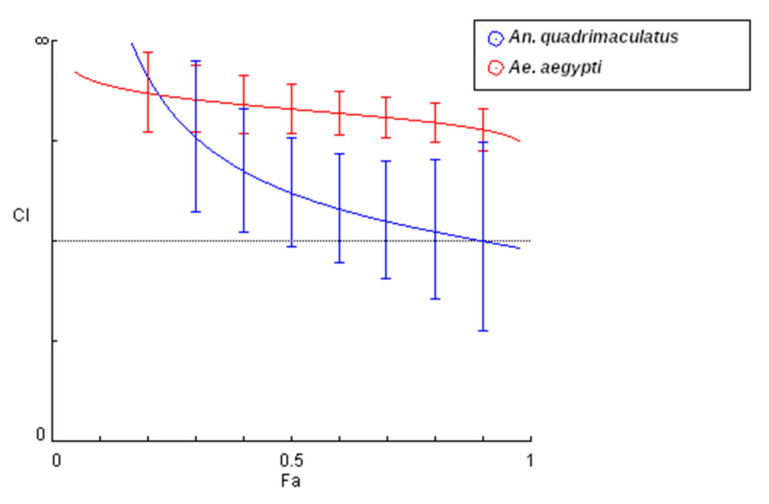
Combination index (*CI*) plot showing the simulated relationship between DL-methionine and *Bacillus thuringiensis* subspecies *israelensis* (BTI) for *Anopheles quadrimaculatus* (blue; n = 4) and *Aedes aegypti* (red; n = 6) exposed for 48 h in glass jars. Error bars are 95% confidence intervals around a mean calculated by sequential deletion analysis (S.D.A.). *CI* less than 1 (below the dotted line) indicates synergism. Lethality fraction (*f*_a_) affected as a proportion on the x-axis. Simulated and plotted using the software Compusyn [30].

**Figure 5:**
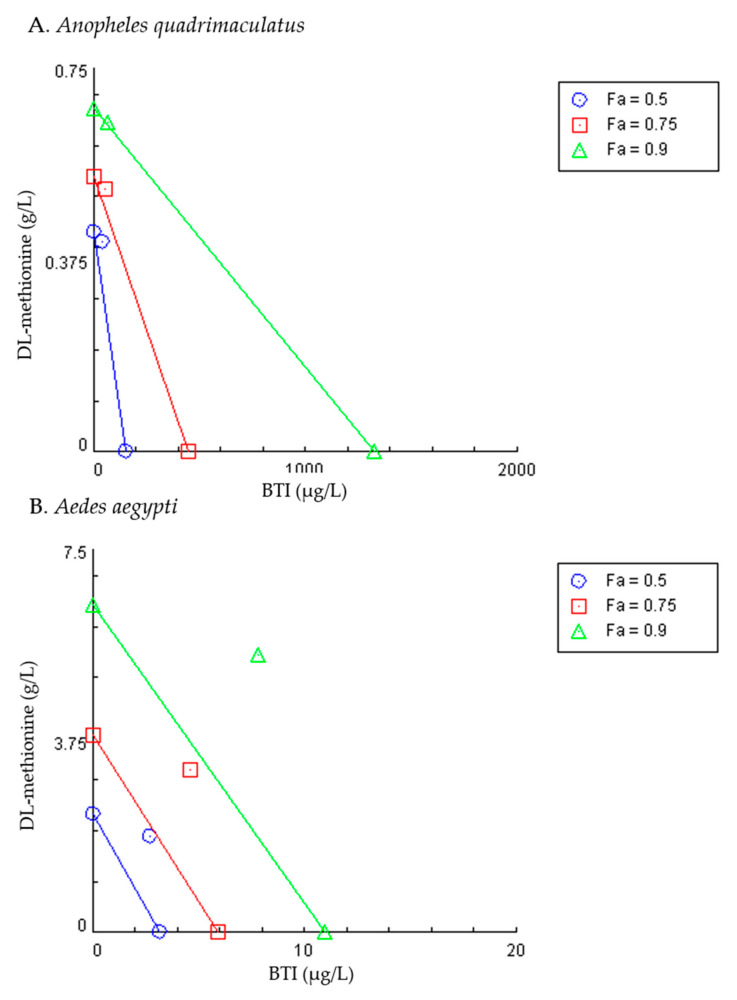
Isobolograms for *Anopheles quadrimaculatus* ((**A**), n = 4) and *Aedes aegypti* ((**B**), n = 6) for DL-methionine and *Bacillus thuringiensis* subspecies *israelensis* (BTI) alone and in combination exposed for 48 h in glass jars. Each line and set of symbols represent the lethality fraction (*f*_a_) affected by the concentration. The concentration of DL-methionine on the y-axis and BTI on the x-axis. A line is drawn between the LC_50_ of each active ingredient on its respective axis. The position of the data point for the combined treatment (data point not on either axis) indicates synergistic (below the line), additive (on the line) or antagonistic (above the line) effects. Plotted using the software Compusyn [30].

In *An. quadrimaculatus,* the relationship between the two AI was complementary. At the highest concentration tested of 4.0X the LC_50_ of each AI, for the actual experimental points the *CI* value was lower than 1.0 and between 0.3 and 0.7, indicating synergism (Table 3, Figure 4). At lower concentrations, the two AIs were varying levels of antagonistic with *CI* above 1. The S.D.A. of the simulated data revealed that although *CI* fell below 1 at an effect level of 0.95 (95% mortality), this was indicative of a nearly additive relationship (*CI* of 0.9–1.10). Furthermore, the isobologram depicted in Figure 5 showed that at all three effect levels (50, 75, and 90% mortality) the relationship was additive. The concentration reduction index in Table 4 shows that it is possible to achieve greater than 80% mortality with an 8-fold reduction in BTI concentration when the combination is utilized.

In contrast, a complementary relationship was not detected at the concentrations tested when the combination of DL-methionine and BTI was used against *Ae. aegypti*. At all concentrations tested, *CI* were above 1, indicating varying levels of antagonism, meaning when combined, DL-methionine and BTI reduced the overall effectiveness of each individual AI with this mosquito species (Table 3). The S.D.A. analysis (Figure 4), isobologram (Figure 5), and concentration reduction indices (Table 4) support this result.

## 4. Discussion

Larvicides are an important component of any integrated vector management program. However, non-target effects, resistance and registration cancellation make the use of historically effective larvicides difficult. The aim of this study was to determine if the relationship between DL-methionine and BTI would result in an enhanced larvicidal effect. Using two mosquito species in different genera (*An. quadrimaculatus* and *Ae. aegypti*), we demonstrated the effectiveness of each AI alone and in combination.

Prior to testing the combined larvicide, we obtained the lethal concentration values for the larvae of each mosquito species with both DL-methionine and BTI. With DL-methionine, we found the LC_50_ value for 2nd instar larvae of *Ae. aegypti* after 48 h to be 3.34 g/L (2.869–3.748 g/L). This value was similar to that reported for *Ae. aegypti* in a previous study by Richardson et al. (3.41 g/L) [20]. These values are 4-fold higher than the value obtained by Weeks et al. (0.81 g/L) [19], and used in this study when testing DL-methionine against *An. quadrimaculatus*. This indicates that DL-methionine was much less toxic to *Ae. aegypti* than *An. quadrimaculatus*. In a previous study, *An. quadrimaculatus* were 10-fold more sensitive to DL-methionine than other mosquito species tested, i.e., *Aedes albopictus* Skuse and *Culex tarsalis* Coquillett [19]. We demonstrated in this study, as well as an earlier study, that methionine can be used as an effective larvicide against *Anopheles* spp. [19]. Furthermore, as an essential amino acid, it is unlikely that insects will develop resistance to methionine.

With regards to BTI sensitivity, we found the LC_50_ to be 87.63 µg/L (45.93–167.70 µg/L) for 3rd instar *An. quadrimaculatus* and 5.02 µg/L (4.23–5.74 µg/L) for 2nd instar *Ae. aegypti*. This difference in lethal concentration values between the two species shows the varied sensitivity to BTI, with *Ae. aegypti* having a much higher sensitivity to BTI than *An. quadrimaculatus*. In general, *Anopheles* spp. larvae have a lower sensitivity to BTI, which is likely due to their positioning in the water column [19]. Due to *Anopheles* spp. having short siphons, they orient themselves parallel to the water surface to feed while *Aedes* spp. orient themselves perpendicular to the water surface [31]. As BTI toxins have a high settling rate they sink to the bottom of the water column unless formulated to float, where the toxins are less accessible to and therefore less effective as a larvicide against *Anopheles* spp. [8,32]. Although further research is needed in the area, due to the efficacy of DL-methionine against *An. quadrimaculatus* demonstrated herein, as well as in Weeks et al. [19], we assume that DL-methionine is being ingested by the larvae of this species and is therefore within the feeding range near the water surface. Therefore, DL-methionine maybe a more useful tool when specifically controlling *Anopheles* spp. mosquitoes than BTI, although this is likely to depend upon how the AIs are formulated.

In this study, in the range of concentrations tested, we also found that a complementary relationship existed between the two AIs in *An. quadrimaculatus* at higher concentrations and effect levels but not in *Ae. aegypti* at any concentration tested. According to the observed data, synergism occurred between DL-methionine and BTI at the highest concentration tested of 4.0X the LC_50_ of each AI when used against *An. quadrimaculatus*. At lower concentrations, the observed data revealed that the relationship between DL-methionine and BTI was antagonistic. In the simulation, the relationship was antagonistic at low effect levels of 5% to 70% mortality and additive at effect levels ≥75%. The isobologram indicated an additive relationship at the LC_50_. Other researchers have noted the potential for biased *CI* estimates at very low or very high effect levels [27,33]. Consequently, the synergism reported herein at the highest tested concentration with an observed effect level of 100% mortality should be considered with caution. In contrast, antagonism was detected at all concentrations and effect levels when DL-methionine and BTI were tested against *Ae. aegypti.* A complementary relationship between BTI and methionine could enable the creation of an enhanced larvicide with improved efficacy against *Anopheles* spp. in comparison to the AIs individually. This combined larvicide could be placed in natural bodies of water where mosquitoes develop. A recent study has shown that DL-methionine is equally as effective in different types of water with varying physiochemical qualities [20]. The combination of two AIs can assist in resistance management in insects by exposing those insects with and without resistant genes to two different AIs, which lowers their chances of surviving and perpetuating resistance. Additionally, as the combination could allow for the reduction in the concentration of BTI needed by greater than 8-fold to achieve control of *Anopheles* species (according to the concentration reduction indices in Table 4) then there could be direct economic benefits. As DL-methionine is used globally as a livestock and aquaculture nutritional supplement [34], the industrial economies of scale favor its commercial use in mosquito control.

While the results above suggest that a combined larvicide would be efficacious for management of *Anopheles* species compared to BTI alone the effect of this combination on non-target organisms is currently unknown and would need to be investigated further. However, based on what is known about the mode of action of methionine, application of a combined larvicide with lower concentrations of BTI could be beneficial in sensitive ecosystems as while DL-methionine is not mosquito-specific, its action is limited on those insects with an alkaline midgut. Chironomid midges are an important part of the food web and are negatively affected by BTI [12]. As chironomid midges have a neutral midgut pH, they would be unlikely to be affected by DL-methionine [35], potentially resulting in less impact on the predators that consume these organisms.

BTI has shown synergism with other AIs that collectively contribute towards the desired bottom-line goal of enhanced larvicidal activity while simultaneously providing an elective approach that prevents larval resistance. For example, Tetreau et al. [36] combined BTI with synthetic chemicals and found that deltamethrin was the only chemical that significantly enhanced the toxicity of BTI against *Ae. aegypti*. In contrast to that study, the other class of effective BTI synergistic reagents are biologics. For example, Sreshty et al. [37] found that *L. sphaericus*, a closely related species to BTI, has synergistic properties when combined with BTI that can be used to control *Ae. aegypti* and *Culex quinquefasciatus* Say larvae. Additionally, BTI was found to have synergistic properties against *Anopheles stephensi* Liston larvae when combined with ethanolic extracts of the herb *Andrographis paniculata* [38]. BTI and *An. paniculata* extract was found to be 52-fold more toxic than BTI alone against mosquito larvae [38]. 

We made the decision to match the mosquito larvae of the two species based on size rather than instar as we felt this would be a more accurate representation of how they would be affected by the concentration of the larvicides. As different instars were used per species (3rd instar for *An. quadrimaculatus* and 2nd for *Ae. aegypti*), this could be a possible limitation in our study for accurately assessing and comparing the synergistic properties between the two species. A second limitation is that we used different water types for each species as we matched the rearing procedures for the colony to avoid environmental stress that could cause mortality. This difference should be kept in mind when directly comparing the results between the species. However, it is relevant to note that our previous research has indicated that within the same species water type did not significantly affect sensitivity to DL-methionine [20]. Due to known differences in sensitivity of the two AIs in different instars and species as well as potential differences due to variation in strain sensitivity, future work should include the testing of this combined larvicide on additional instars, mosquito species, and different colonies. 

## 5. Conclusions

In this study, we found that when used alone BTI was much less efficacious against *An. quadrimaculatus* than *Ae. aegypti* larvae. Furthermore, when DL-methionine and BTI were combined, an additive effect occurred at higher concentrations in *An. quadrimaculatus*. However, antagonism was found when this same combination was used against *Ae. aegypti.* Our findings contribute to the growing demand to develop environmentally sustainable pesticides that also can combat insect resistance to current pesticides. Our research indicate that DL-methionine is effective alone and a complementary relationship exists between DL-methionine and BTI against *An. quadrimaculatus,* which creates the potential for this AI to be used alone and in combination in newly developed and marketed pesticides to help control *Anopheles* species. As *Anopheles* spp. are vectors of malaria, developing a novel pesticide could be of crucial benefit to public health.

## Figures and Tables

**Table 1:** Lethal concentration (LC) values of *Anopheles quadrimaculatus* 3rd instar larvae and *Aedes aegypti* 2nd instar larvae when exposed to DL-methionine (g/L) and *Bacillus thuringiensis* subspecies *israelensis* (BTI; µg/L) for 48 h in glass jars to achieve 50% (LC_50_), 90% (LC_90_), and 99% (LC_99_) mortality. LC values calculated by probit analysis with PoloPlus (PoloPlus 1.0; LeOra Software, El Cerrito, CA, USA). See Supplementary Materials (Tables S1–S3) for mortality values.

Mosquito Species	n (N)	Treatment Slope ± SE	Natural Response Slope ± SE	LC_50_	LC_90_	LC_99_	χ^2^
**Methionine (g/L)**							
*Anopheles quadrimaculatus*	NA	NA	NA	0.81 (0.77–0.85) ^a^	1.17 (1.10–1.26) ^a^	NA	NA
*Aedes aegypti*	4 (1198)	3.903 ± 0.297	0.040 ± 0.013	3.34 (2.87–3.75)	7.10 (6.34–8.25)	13.17 (10.84–17.53)	31.19
**BTI (µg/L)**							
*Anopheles quadrimaculatus*	1–5 (1300)	1.256 ± 0.078	0.069 ± 0.015	87.63 (45.93–167.70)	917.79 (412.09–3664.95)	6228.60 (1891.70–59,034.00)	125.16
*Aedes aegypti*	1–7 (1579)	3.669 ± 0.208	0.031 ± 0.010	5.02 (4.23–5.74)	11.21 (9.60–13.97)	21.60 (16.71–32.37)	109.41

LC values with 95% inverse confidence intervals in parentheses. Chi-squared (χ^2^) values are listed. NA = not available. n = number of replicates. N = number of mosquitoes. ^a^ data extracted from Weeks et al. [19] and units converted.

**Table 2:** Concentration response analysis of DL-methionine, *Bacillus thuringiensis* subspecies *israelensis* (BTI), and their combination on *Anopheles quadrimaculatus* (n = 4) and *Aedes aegypti* (n = 6) exposed for 48 h in glass jars. Slopes of the median effects plots with standard errors and lethal concentrations to 50% mortality (LC_50_) for the combination experiment. Calculated using the software Compusyn [30]. See Supplementary Materials (Tables S4 and S5) for mortality values.

Species	Larvicide	Slope (±SE)	LC_50_	*R*
*An. quadrimaculatus*	BTI	1.01054 (0.15796)	150.905 µg/L	0.96525
	DL-methionine	4.90050 (1.64185)	0.43020 g/L	0.86492
	Combination	4.88237 (1.55613)	44.7912	0.87546
*Ae. aegypti*	BTI	1.77700 (0.15493)	3.19775 µg/L	0.98879
	DL-methionine	2.14708 (0.13018)	2.31365 g/L	0.99453
	Combination	2.06858 (0.14742)	4.59306	0.99247

Combination concentration does not have units. The *R* value is the linear correlation coefficient, which represents the data’s conformity to the mass-action law [27].

**Table 3:** Lethality fraction (*f*_a_) affected by the combined larvicide of DL-methionine and *Bacillus thuringiensis* subspecies *israelensis* (BTI) concentration as a proportion and the calculated combination indices (*CI*) for *Anopheles quadrimaculatus* (n = 4) and *Aedes aegypti* (n = 6) and at each actual concentration tested in a 48-h exposure in glass jars. *CI* calculated using the software Compusyn and synergism/antagonism categories provided according to Chou and Martin [30].

Species	Concentration (X)	*f_a_*	*CI*	Synergism/Antagonism Category
*An. quadrimaculatus*	0.25	0.17808	1.30160	Moderate antagonism
	0.50	0.43880	1.35974	Moderate antagonism
	1.00	0.83556	1.46738	Antagonism
	2.00	0.94815	2.14648	Antagonism
	4.00	1.00000	0.44928	Synergism
*Ae. aegypti*	0.25	0.19982	1.50795	Antagonism
	0.50	0.35457	2.00546	Antagonism
	1.00	0.77275	1.57021	Antagonism
	2.00	0.91667	1.72374	Antagonism
	4.00	0.98639	1.32453	Moderate antagonism

Units for concentration of the combination were X times the LC_50_ for each active ingredient.

**Table 4:** Concentration reduction index (*CRI*) of DL-methionine and *Bacillus thuringiensis* subspecies *israelensis* (BTI) for *Anopheles quadrimaculatus* (n = 4) and *Aedes aegypti* (n = 6). Assays were conducted for 48 h in glass jars. Concentrations of each active ingredient to achieve the lethality fraction (*f*_a_) of the combined larvicide and the *CRI* for each active ingredient for each species calculated using the software Compusyn [30].

		Concentration		CRI	
Species	f_a_	DL-Methionine g/L	BTI µg/L	DL-Methionine	BTI
*An. quadrimaculatus*	1.00000	7.21160	1323 × 10^9^	2.22580	373317
	0.94815	0.77843	4270.430	0.48051	15.2980
	0.83556	0.59941	968.045	0.74002	8.61598
	0.43880	0.40913	110.645	1.01020	2.70388
	0.17808	0.31487	24.9960	1.55490	1.51868
*Ae. aegypti*	0.98639	17.0090	35.6172	1.27313	1.85506
	0.91667	7.06859	12.3281	1.05817	1.28417
	0.77275	4.09128	6.36742	1.22493	1.32655
	0.35457	1.75039	2.28269	1.04814	0.95112
	0.19982	1.21244	1.46474	1.45203	1.22062

*CRI* < 1 is unfavorable concentration reduction, *CRI* > 1 is favorable concentration reduction, *CRI* = 1 no concentration reduction.

## Supplementary Materials

The following are available online at https://www.mdpi.com/2075-4450/11/12/880/s1, Table S1: Mortality of *Aedes aegypti* 2nd instar larvae when exposed to *Bacillus thuringiensis* subspecies *israelensis* (BTI; µg/L) for 48 h in glass jars. Average mortality and standard errors calculated when three of more replicates were completed. Number of replicates, as well as jars and mosquitoes tested provided, Table S2: Mortality of *Anopheles quadrimaculatus* 3rd instar larvae when exposed to *Bacillus thuringiensis* subspecies *israelensis* (BTI; µg/L) for 48 h in glass jars. Average mortality and standard errors calculated when three of more replicates were completed. Number of replicates, as well as jars and mosquitoes tested provided, Table S3 Mortality of *Aedes aegypti* 2nd instar larvae when exposed to DL-methionine (g/L) for 48 h in glass jars. Average mortality and standard errors calculated. Number of replicates, as well as jars and mosquitoes tested provided, Table S4: Concentration response analysis of DL-methionine, *Bacillus thuringiensis* subspecies *israelensis* (BTI), and their combination on *Aedes aegypti* (n = 6, 12 jars) exposed for 48 h in glass jars (10 to 15 mosquitoes per jar). Average mortality (%) and standard errors provided, Table S5: Concentration response analysis of DL-methionine, *Bacillus thuringiensis* subspecies *israelensis* (BTI), and their combination on *Anopheles quadrimaculatus* (n = 4, 12 jars) exposed for 48 h in glass jars (10 to 15 mosquitoes per jar). Average mortality (%) and standard errors provided.
