# Supplementary material for: Combining DL-Methionine and Bacillus thuringiensis Subspecies israelensis: Prospects for a Mosquito Larvicide
Source: Insects. 2020 Dec 11;11(12):880. doi: 10.3390/insects11120880 (PMC7764334; doi:10.3390/insects11120880)
Supplement: Supplementary file 1 [file insects-11-00880-s001.pdf]

Table S1. Mortality of *Aedes aegypti* 2nd instar larvae when exposed to *Bacillus thuringiensis* subspecies *israelensis* (BTI; µg/L) for 48 hours in glass jars. Average mortality and standard errors calculated when three of more replicates were completed. Number of replicates, as well as jars and mosquitoes tested provided.

Concentration (µg/L)	Mortality	Standard error	Replicates	Jars	Mosquitoes
0	2.70	1.05	7	28	329
2.4	19.75	4.86	6	24	263
4.8	42.68	5.11	4	16	189
6	97.50	N/A	1	4	40
7.2	58.50	6.71	4	16	190
9.6	86.79	3.70	4	16	194
12	95.12	2.17	7	28	329
24	100.00	N/A	1	4	45

Table S2. Mortality of *Anopheles quadrimaculatus* 3rd instar larvae when exposed to *Bacillus thuringiensis* subspecies *israelensis* (BTI; µg/L) for 48 hours in glass jars. Average mortality and standard errors calculated when three of more replicates were completed. Number of replicates, as well as jars and mosquitoes tested provided.

Concentration (µg/L)	Mortality	Standard error	Replicates	Jars	Mosquitoes
0	7.99	2.98	5	20	241
2.4	0.00	N/A	1	4	46
4.8	0.00	N/A	1	4	58
7.2	2.08	N/A	1	4	48
9.6	8.70	N/A	1	4	46
12	26.80	7.36	5	20	253
24	37.55	5.49	4	16	207
120	40.00	N/A	1	4	45
240	62.29	6.87	4	16	202
1200	100.00	0.00	3	12	154

Table S3. Mortality of *Aedes aegypti* 2nd instar larvae when exposed to DL-methionine (g/L) for 48 hours in glass jars. Average mortality and standard errors calculated. Number of replicates, as well as jars and mosquitoes tested provided.

Concentration (µg/L)	Mortality	Standard error	Replicates	Jars	Mosquitoes
0	3.09	1.29	4	16	201
1	7.89	2.93	4	16	209
2.5	32.05	6.23	4	16	198
5	76.24	3.70	4	16	198
7.5	91.82	1.41	4	16	195
10	97.53	1.20	4	16	197

Table S4. Concentration response analysis of DL-methionine, *Bacillus thuringiensis* subspecies *israelensis* (BTI), and their combination on *Aedes aegypti* (n=6, 12 jars) exposed for 48 hours in glass jars (10 to 15 mosquitoes per jar). Average mortality (%) and standard errors provided.

Active Ingredient	Mortality (%)	Standard error
Negative control (water)	9.82	5.10
DL-methionine (g/L)		
0.835	12.40	6.67
1.670	30.79	9.17
3.340	60.10	11.10
6.680	91.94	5.94
13.360	97.94	0.92
BTI (µg/L)		
1.2	15.43	5.67
2.4	44.18	9.76
4.8	56.03	15.31
9.6	87.25	6.21
19.2	96.70	1.23
Combination MetBTI (times the LC_{50})		
0.25X	19.98	7.00
0.50X	35.46	7.07
1.00x	77.27	3.93
2.00x	91.67	2.85
4.00x	98.64	0.86

Units for concentration of the combination were X times the LC_{50} for each active ingredient (see Table 2).

Table S5. Concentration response analysis of DL-methionine, *Bacillus thuringiensis* subspecies *israelensis* (BTI), and their combination on *Anopheles quadrimaculatus* (n=4, 12 jars) exposed for 48 hours in glass jars (10 to 15 mosquitoes per jar). Average mortality (%) and standard errors provided.

Active Ingredient	Mortality (%)	Standard error
Negative control (water)	14.24	5.56
DL-methionine (g/L)		
0.2025	19.73	9.53
0.4050	30.30	4.29
0.8100	77.36	4.71
1.6200	93.68	1.57
3.2400	100.00	0.00
BTI (µg/L)		
21.875	12.84	1.10
43.750	22.94	4.23
87.500	39.32	5.09
175.000	41.35	9.34
350.000	76.06	13.95
Combination MetBTI (times the LC_{50})		
0.25X	17.81	4.13
0.50X	43.88	9.72
1.00x	83.56	8.61
2.00x	94.82	2.84
4.00x	100.00	0.00

Units for concentration of the combination were X times the LC_{50} for each active ingredient (see Table 2).
